# Formaldehyde Fumigation: Antibacterial Profile and Toxic Effects on Hatching Eggs

**DOI:** 10.3390/toxics13100851

**Published:** 2025-10-09

**Authors:** Pedro Henrique Gomes de Sá Santos, Gabriel da Silva Oliveira, Liz de Albuquerque Cerqueira, José Luiz de Paula Rôlo Jivago, Susana Suely Rodrigues Milhomem Paixão, Márcio Botelho de Castro, Concepta McManus, Vinícius Machado dos Santos

**Affiliations:** 1 Faculty of Agronomy and Veterinary Medicine, University of Brasília, Brasília 70910-900, Brazil; 2 Laboratory of Poultry Science, Federal Institute of Brasília—Campus Planaltina, Brasília 73380-900, Brazil; 3 Institute of Biological Sciences, University of Brasília, Brasília 70910-900, Brazil; 4 Center for Nuclear Energy in Agriculture (CENA), University of São Paulo, São Paulo 13416-000, Brazil

**Keywords:** chicken eggs, microbiology, poultry science, synthetic chemical products, toxicity

## Abstract

**Highlights:**

**What are the main findings of your study?**

- Formaldehyde (FA) fumigation reduced bacterial load on eggshells.
- Increasing FA concentration intensified eggshell microstructural damage and embryonic tracheal lesions.
- Reduced chick weight and impaired chick quality are among the toxic effects of FA fumigation.

**What is the implication or significance of these findings?**

- Fumigation with FA as an antibacterial treatment for hatching eggs poses risks to poultry survival, and consequently, to the efficiency and quality of poultry production.

**Abstract:**

Previous studies have linked formaldehyde (FA) fumigation to significant risks to animal health, highlighting, among other effects, its cytotoxic and genotoxic potential. Literature includes several studies on the use of FA for fumigating hatching eggs, but studies employing in-depth methodological approaches are scarce. As a result, the effects of practices involving this chemical remain insufficiently characterized. The present study aimed to investigate the antibacterial effects and potential toxicity resulting from the fumigation of hatching eggs with FA. The three FA concentrations (2.5, 5, and 10 g/m^3^) exhibit effective antibacterial activity, but this effect does not translate into long-term benefits. FA affected hatchability and demonstrated embryotoxic effects, with repercussions on chicks depending on the concentration used. The overall quality of poultry and the losses from eggs fumigated with FA remain questionable. Despite its efficacy as an egg fumigant, the observed toxicity suggests that its use violates safety standards and should be reconsidered. If its use cannot be avoided, the lowest possible concentrations should be prioritized to minimize toxic effects.

## 1. Introduction

The eggshell is a vital yet temporary structure that serves as the foundation for embryonic development [1]. However, the bacterial community found on the eggshell, acquired either vertically or horizontally, has been associated with adverse prenatal and perinatal outcomes, including increased bacterial contamination of the yolk sac, higher rates of both early and late embryonic death (EED; LED), and reduced hatchability (HI) and chick weight (CW) [2]. Given that some degree of bacterial contamination in hatching eggs is expected, efforts are focused on minimizing it by leveraging the antibacterial effects of sanitizing compounds used in poultry production. Depending on the sanitizing agent employed, the benefits of bacterial control may be offset by detrimental effects on poultry survival due to the compound’s inherent toxicological properties. Formaldehyde (FA), the primary agent used internationally for fumigating hatching eggs, exemplifies this issue [3].

Despite the satisfactory productivity observed with the use of FA in egg fumigation, the actual health status of hatched chicks remains questionable, as these may experience internal, non-visible damage, a condition that poses a significant risk to their survival [4]. Research on the use of FA in poultry production requires further investigation, especially in light of the growing interest in practices aimed at controlling the bacterial load on eggshells, the need to elucidate potential harm to poultry, and the importance of identifying and monitoring the risk factors associated with such damage. Based on this understanding, it is possible to promote changes and implement interventions to mitigate adverse impacts, particularly those related to toxicity. Detailed and simultaneous evaluations of the antibacterial efficacy of hatching egg fumigation with FA and its toxicological consequences for poultry at the histological and genetic levels, under commercial conditions, remain insufficient. Thus, this study aimed to evaluate the antibacterial and toxic effects of fumigation of hatching eggs with FA.

## 2. Materials and Methods

**Figure 1:**
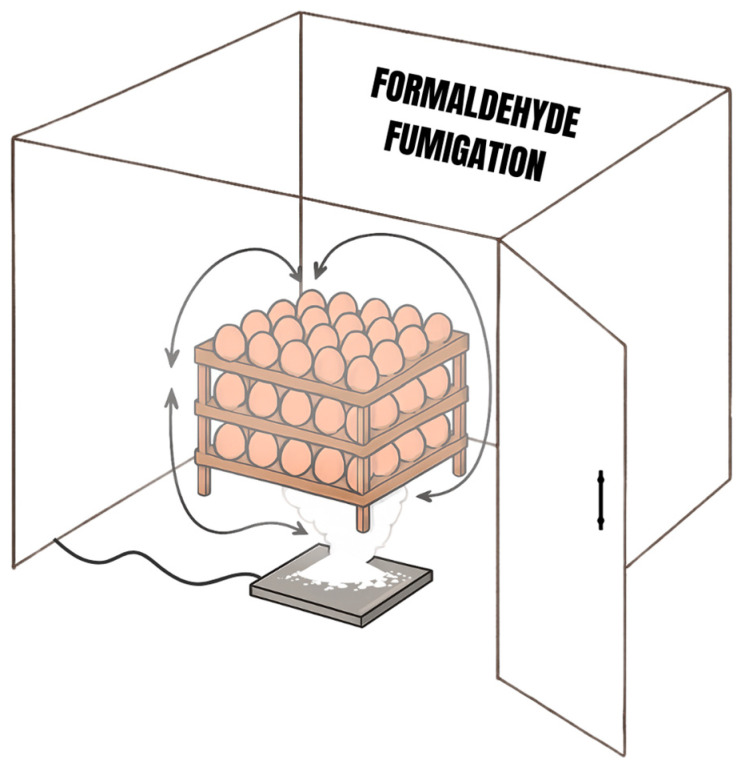
Illustration of the fumigation process of hatching eggs with formaldehyde (FA).

The study was conducted in accordance with the Ethical Principles for Animal Research and was approved by the Ethics Committee on Animal Use of the University of Brasília (Protocol No. 61/2022). Hatching eggs from 51-week-old Cobb broiler breeders were fumigated with FA between 20 and 50 min post-collection (Figure 1), in accordance with the protocol established by a commercial poultry farm, as detailed in Table 1. The commercial poultry farm routinely uses FA fumigation at a concentration of 5 g/m^3^, using 91% paraformaldehyde (Ercros, Barcelona, Spain) as the source. Reports on the use of FA for fumigating hatching eggs indicate concentrations ranging approximately from 2 to 13 g/m^3^ [5,6,7,8,9]. Based on USDA [10] and Mineki and Kobayashi [11], the internal quality parameters of the hatching eggs evaluated in this study (n = 20), namely, the Haugh unit (84.24 ± 3.91) and yolk index (0.39 ± 0.02), are indicative of fresh eggs with excellent quality.

Six eggs from each treatment were individually placed in sterile bags containing 0.1% peptone water and manually massaged to extract the bacterial load from the eggshell surface, following the method described by Vale et al. [12]. This water was analyzed to determine the total aerobic mesophilic bacteria (TAMB) and Enterobacteriaceae (ENT) present on eggshell surface. The resulting solution from each bag was serially diluted, and 0.1 mL from each sample was pipetted onto the surface of Petri dishes containing count agar (Laborclin, Paraná, Brazil) and violet red bile glucose agar (Laborclin, Paraná, Brazil). The plates were incubated at 36 °C for 48 h. The colonies were counted, and the results were log_10_ transformed.

Fumigated and non-fumigated eggshells (eight/treatment) were prepared for microstructural analysis [13], and, after preparation, they were subjected to metalization and examined via a JEOL JSM-7001F scanning electron microscope (Jeol Ltd., Akishima, Tokyo, Japan) at a standard magnification of up to ×4000. Morphological, textural, and structural modifications of the eggshells were characterized from the micrographs.

Eggs that were not subjected to bacteriological and microscopic analyses were stored for 24 h at approximately 20 °C and 50% relative humidity and then transported under refrigeration to a commercial hatchery, where they remained under the same conditions for an additional 24 h. After this period, the eggs were weighed, placed in trays (84 eggs/tray) and incubated in a multi-stage setter with a capacity of 120,000 eggs. They remained here for 18 days, during which time they were turned continuously every hour. A second weighing followed this process. On the 19th day of incubation, the eggs were transferred to a commercial hatcher with a capacity of 128,000 eggs, where they remained for an additional 3 days. During the setting period, the temperature was maintained between 37.5 °C and 38 °C, whereas in the hatcher, it was stable between 36.9 °C and 36.5 °C. In both stages, the relative humidity was consistently maintained at approximately 50%. No fumigation with FA was performed in either the setter or the hatcher. Vaccination was not performed in ovo, only after hatching. After the incubation process, the percentages of egg weight loss (EWL), HI, contaminated eggs (CE), and mortality, including EED, intermediate death (IED), and LED, were recorded. The formulas used to obtain these results are detailed in the study by Oliveira et al. [5]. Additionally, the weight in grams of all hatched chicks was recorded, and the quality of 40 chicks was assessed based on reflexes, navel, legs, and beak, according to the criteria described by Boerjan [14].

Samples were prepared in triplicate per treatment to count TAMB and ENT from the yolk sac [15]. Each solution was made by homogenizing 1 g of a composite sample (yolk sacs from two embryos on day 18 of development) in 9 mL of 0.1% peptone saline solution. The resulting solution was serially diluted, and 0.1 mL from each sample was pipetted onto the surface of Petri dishes containing count agar (Laborclin, Paraná, Brazil) and violet red bile glucose agar (Laborclin, Paraná, Brazil). The plates were incubated at 36 °C for 48 h. Colony-forming units (CFUs) were counted, and the data were log_10_-transformed.

Six embryos from each treatment group were euthanized by cervical dislocation on day 18 of incubation, and tracheal samples were collected for histological analysis. After collection, the samples were fixed in a 10% formalin solution (pH 7.0), embedded in paraffin, and stained with hematoxylin and eosin according to the protocol described by Oliveira et al. [4], which was adapted from Hayretdağ and Kolankaya [16]. Through histological analysis, tracheal lesions in embryos were morphologically evaluated and classified according to severity as absent (−), mild (+), moderate (++), or severe (+++) across four categories: epithelial cell necrosis (ECN), goblet cell hyperplasia (GCH), lymphocytic inflammation (LI), and epithelial cell degeneration (ECD).

Blood samples were collected from six chicks per treatment group by puncturing the metatarsal vein using a 26G insulin syringe (1 mL/U100) immediately after hatching for the micronucleus test [17]. A drop of blood was placed directly onto glass slides, and blood smears were prepared in duplicate for each sample. The smears were fixed in absolute methanol for 10 min and then stained with 5% Giemsa solution for an additional 10 min. The stained slides were analyzed under a light microscope (Zeiss Primo Star) at 400× magnification. The frequency of alterations was counted in 1000 erythrocytes. Upon reaching this number per smear, the occurrence of cells with micronuclei and/or nuclear abnormalities, including binucleated, notched, lobed, blebbed, kidney-shaped, anucleated, pyknotic, and apoptotic cells was recorded [18]. In some commercial hatcheries, FA fumigation is routinely applied at multiple stages of incubation as a preventive measure to control bacterial contamination. Therefore, six additional chicks, originating from eggs incubated in a different setter where routine FA fumigation was applied until hatching, were also evaluated to determine whether any differences existed in the results of the micronucleus test between eggs not exposed to FA during incubation (FA-I, FA-II, and FA-III) and those exposed (FA-IN). Fumigation during incubation was carried out using a FA solution (36.5–38%) diluted in autoclaved water. Inside the setter, the solution was prepared at a ratio of 1:4 (FA:water), whereas in the hatcher a ratio of 2:3 (FA:water) was used, with the liquid placed in open containers.

Statistical analyses were performed via GraphPad Prism 5 (https://www.graphpad.com accessed on 23 July 2025) or SAS software version 9.4 (SAS Institute Inc., Cary, NC, USA). Data were compared among treatments using analysis of variance followed by Tukey’s test (PROC GLM) or the Kruskal—Wallis test (PROC NPAR1WAY) for normally and non-normally distributed data, respectively. Differences between treatments were considered significant at *p* < 0.05. Correlation analyses between microbiological variables and incubation performance were also conducted via PROC CORR.

## 3. Results and Discussion

For TAMB, all fumigated groups (FA-I, FA-II, and FA-III) presented counts <10 CFU on the eggshell, showing a significant effect (*p* < 0.05) when compared with the control group (Table 2). In the yolk sacs, fumigation did not have the same effect. Although a slight numerical reduction in this population was observed in the treated groups compared with the control, the difference was not statistically significant. ENT counts, both on the eggshell and in the yolk sacs, were already <10 CFU in the control group and remained at that level in the treated groups. According to dos Santos et al. [9], fumigating hatching eggs with FA reduces the TAMB load on the eggshell (ETAMB) surface by more than 50%. Despite prior knowledge of FA’s ability to reduce bacterial contamination on eggshell surfaces, its ineffectiveness in decreasing contamination in the yolk sac has also been reported [4], showing a positive correlation with contamination [2]. Furthermore, FA limitation in maintaining low bacterial levels on the shell after fumigation has also been documented [19], which may explain the lack of effect on TAMB counts in the yolk sac (YTAMB) observed in this study. It is suggested that the ETAMB may have increased after the sample collection period for bacteriological analysis and given the positive correlation (R = 0.52) between ETAMB and YTAMB (Table 3), it may have negatively influenced YTAMB.

**Figure 2:**
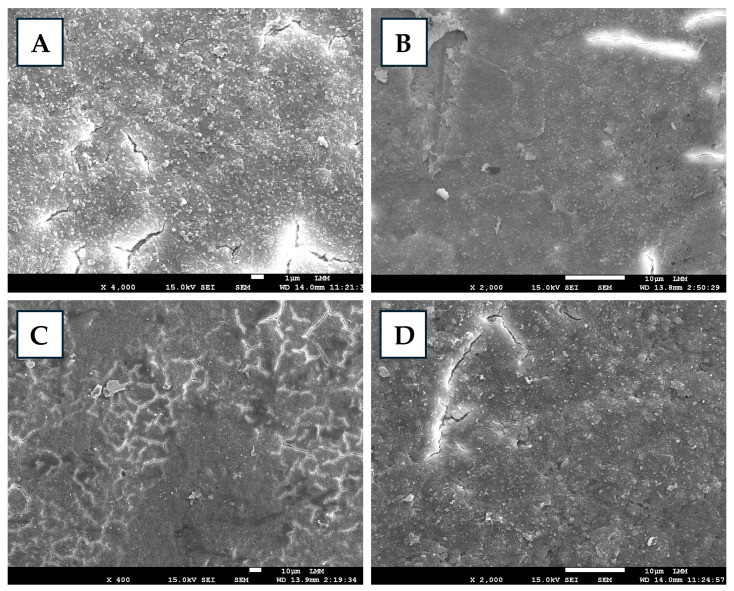
Scanning electron micrographs of eggshells fumigated with or without formaldehyde (FA). Control (**A**), FA fumigation at (**B**) 2.5 g/m^3^—FA-I, (**C**) 5 g/m^3^—FA-II, and (**D**) 10 g/m^3^—FA-III.

The analysis of the electron micrographs revealed that, in the eggshells from the control treatment, the surface exhibited relatively smooth areas interspersed with rough regions and tiny microcracks, where small natural granules were distributed (Figure 2). These microvariations were consistent with the natural morphology and showed no signs of fragmentation. With FA-I fumigation, initial changes in the mineral matrix were already observed, indicating signs of structural weakening. The surface exhibited small scattered irregularities, microgranulations distributed throughout the shell matrix, slight roughness, and minor superficial cracks. In FA-II fumigation, these alterations became more pronounced, with incipient areas of demineralization and partial loss of surface uniformity. A network of fine grooves and cracks forming nearly branched patterns was observed, while small loose particles were distributed heterogeneously. In FA-III fumigation, marked erosion and more evident demineralization were observed, conditions that increase susceptibility to contamination and compromise the mechanical strength of the eggshell. Microcracks and superficial fissures were unevenly distributed across the surface, leading to the detachment of small fragmented particles and an overall irregular texture. Overall, the increase in FA concentration progressively intensified the loss of mineral density in the eggshell. Significant alterations in eggshell integrity resulting from FA fumigation have been previously reported [4,20], confirming its toxicity to the shell and increasing the risk of negative consequences for embryonic development.

A tendency for increased EWL was observed with FA-III fumigation. This difference was not statistically significant among the treatments (Table 4), indicating that the observed eggshell damage was insufficient to cause differences in this variable. Among the different levels of FA fumigation, a dose-dependent pattern was observed for HI and EED. Both variables were negatively correlated (*r* = −0.87) (Table 3). These findings suggest that FA-III may negatively affect embryonic development during the early stages of incubation. In contrast, FA-II may have a variable effect on embryonic development depending on the fumigation conditions, and its use requires caution, as it does not ensure HI and may increase EED. Thus, higher concentrations of FA may have an adverse effect on embryonic development during the initial stages of incubation. A significant EED in eggs fumigated with FA was also reported by Baylan et al. [21] and Oliveira et al. [4], who suggested that these effects are related to the toxicity of the compound to eggshell structures and residual contact with the embryo, given its highly toxic and multi-target nature. Amoah et al. [22] fumigated hatching eggs with 30, 40, or 50 mL of 40% formalin with 20 g of potassium permanganate crystals and also observed that HI decreased as the volume of formalin increased.

The CE did not differ among the treatments, with an average of 0.60%. Correlation analysis revealed that the CE was negatively correlated (*r* = −0.54) with HI (Table 3), suggesting that contamination contributed to the variation in HI rates. Moreover, the CE was positively correlated (*r* = 0.67) with the occurrence of YTAMB, which, in turn, was positively correlated (*r* = 0.52) with ETAMB. These results indicate that the contamination pathway, from the eggshell to the embryo, constitutes a sequence of events that may represent a determining factor for the variations observed in HI rates.

FA-III significantly reduced (*p* < 0.05) CW compared to the control, whereas FA-I and FA-II did not differ from the control or from FA-III (Table 5). For chick quality (CQ), a similar pattern was observed but was restricted to the treated groups. Chicks from FA-I presented better scores than those from FA-III (*p* < 0.05), whereas all the treatments remained statistically similar to the control. These results indicate that the use of FA-III is detrimental to CW and, independently, also compromises CQ compared with FA-I. FA-II, in turn, may exhibit a variable effect on both CW and CQ, depending on the fumigation conditions. The toxic effect of FA on the weight of developing poultry appears to be more prevalent than that of synthetic and natural treatments, and it warrants special attention. Previous studies associated FA with underweight and underdeveloped embryos, as well as with low-weight chicks [4,23,24]. FA was also responsible for reducing CQ, as evidenced by an increase in abnormalities in regions such as the navel area, legs, eyes, beak, yolk, and reflexes [25]. FA continues to exhibit embryotoxicity, with effects that are reflected in the postnatal development of poultry.

**Figure 3:**
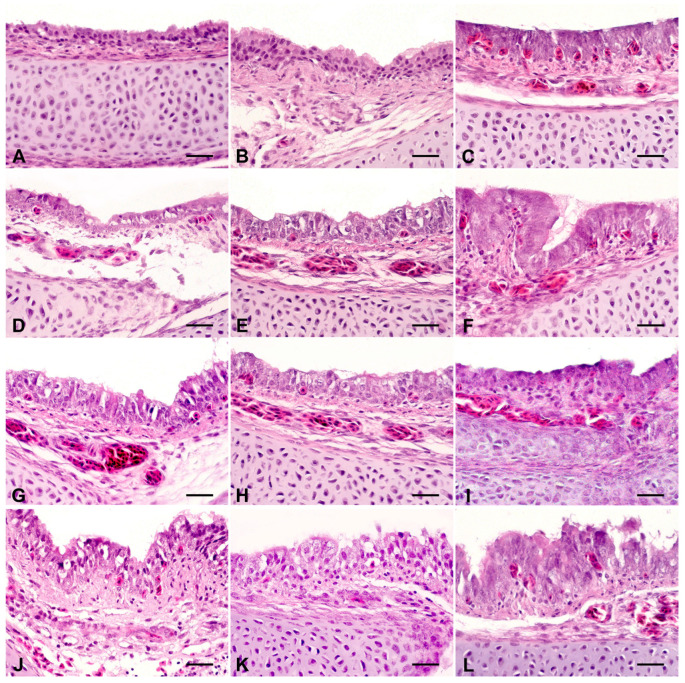
Histological evaluation of the trachea. Hematoxylin and eosin stain, scale bar = 25 µm. Control: (**A**–**C**) showed no morphological changes. FA-I^1^: (**D**) epithelial cell degeneration (ECD); (**E**) goblet cell hyperplasia (GCH); (**F**) mononuclear inflammatory infiltrate in the mucosa (LI). FA-II^1^: (**G**) epithelial cell degeneration (ECD); (**H**) goblet cell hyperplasia (GCH); (**I**) mononuclear inflammatory infiltrate in the mucosa (LI). FA-III^1^: (**J**) epithelial cell degeneration (ECD); (**K**) goblet cell hyperplasia (GCH); (**L**) mononuclear inflammatory infiltrate in the mucosa (LI). ^1^Formaldehyde (FA) fumigation at 2.5 g/m^3^ (FA-I), 5 g/m^3^ (FA-II), and 10 g/m^3^ (FA-III).

Based on histological micrographs and the mean severity scores (−, +, ++, +++), ECN was not observed in any of the groups (Figure 3; Table 6). GCH showed a dose-dependent pattern, with mild effects on FA-I and FA-II and moderate effects on FA-III. The LI, which was absent in the control group, was observed only in FA-III, with mild intensity. FA-I and FA-II revealed no visible signs of this lesion. ECD was one of the most consistent findings among the treated groups, being absent only in the control group and present at moderate intensity in all the fumigated groups. These findings suggest that fumigation with FA, especially at relatively high concentrations, promotes cumulative morphohistological alterations in the embryonic trachea, notably characterized by GCH and ECD. Similar findings have been previously reported in histological studies involving embryos from eggs fumigated with FA, which described shortening and loss of cilia in the tracheal epithelium, cytoplasmic vacuolization, mitochondrial swelling, and disruption of mitochondrial cristae, indicating deleterious effects on the cellular and functional integrity of the respiratory epithelium [16]. Additionally, lesions consistent with GCH and LI have also been described [4], supporting the findings of the present study. Maharjan et al. [26] reported that chicks hatched from eggs fumigated inside hatchers with 37% FA-based products exhibited desquamation of the tracheal columnar epithelium. The tracheal alterations observed in the embryos indicate an early impairment of the functional integrity of the respiratory epithelium, which may persist in newly hatched chicks, predisposing them to increased susceptibility to respiratory infections and difficulties in adaptation during the first days of life, with possible repercussions extending into adulthood. These results further reinforce the relationship between FA fumigation of hatching eggs and the occurrence of respiratory problems in poultry.

**Figure 4:**
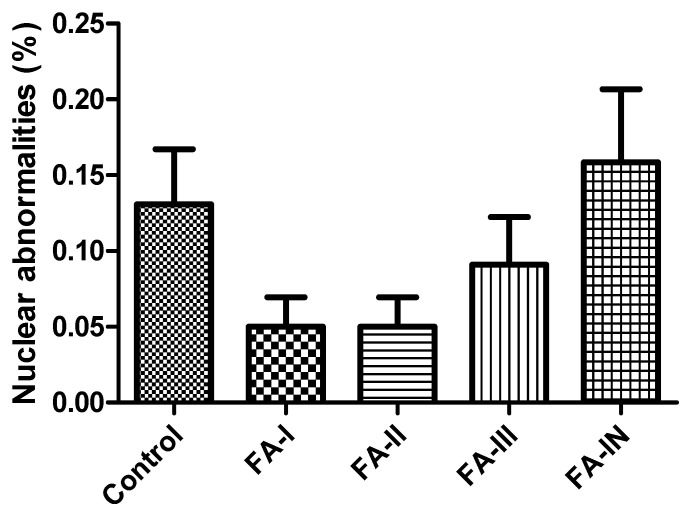
Frequency of nuclear abnormalities in erythrocytes of chicks hatched from eggs subjected to different levels of formaldehyde (FA) fumigation. FA fumigation was performed at 2.5 g/m^3^ (FA-I), 5 g/m^3^ (FA-II), and 10 g/m^3^ (FA-III), and FA was fumigated during incubation (FA-IN).

**Figure 5:**
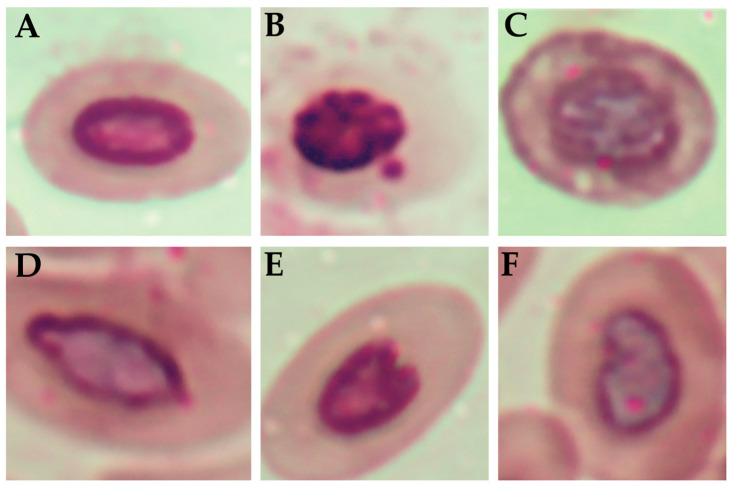
Nuclear abnormalities in erythrocytes of chicks hatched from eggs subjected to different levels of formaldehyde (FA) fumigation. (**A**) Normal nuclei; (**B**) Micronucleated; (**C**) Apoptotic nuclei; (**D**) Blebbed nuclei; (**E**) Notched nuclei; (**F**) Kidney nuclei.

FA fumigation at concentrations ranging from 2.5 to 10 g/m^3^, as well as its administration during incubation, enabled the detection of genetic alterations in newly hatched chicks through the micronucleus test, resulting from chemical exposure (Figure 4 and Figure 5). Statistical analysis revealed that the control group presented a frequency of nuclear abnormalities similar to those of the other groups, and comparable frequencies were also observed among the different fumigation levels, suggesting a lack of direct linear association between the concentration of the fumigant and the severity of nuclear damage. The highest mean frequency of nuclear abnormalities was observed in the FA-IN fumigation, a scenario distinct from fumigation carried out prior to incubation. However, this difference was not statistically significant in terms of genotoxic effects measured by the test. The lack of significant genetic damage at the tested concentrations may be attributed not only to the fumigation levels applied but also to a possible correction or repair of such damage by the chicks themselves before blood collection. The samples were collected immediately after hatching. However, in the treatments where FA was fumigated only during pre-incubation, there was a considerable interval between the embryo’s exposure to the agent and blood collection, which may have led to an underestimation of short-term DNA damage. This hypothesis is supported by the general understanding that hematopoietic renewal can lead to the replacement of altered erythrocytes over time [27]. This may also explain why FA-IN fumigation did not differ significantly from the other groups. The fumigation occurred shortly before blood collection but the genotoxic effects that occurred earlier during embryonic development were likely not detected in the blood samples, as cells damaged at previous stages of incubation may have already undergone repair. The data do not indicate significant genotoxicity in poultry under the tested conditions, but further investigations using more specific protocols or more sensitive methods are necessary to fully clarify its potential genotoxic effects. Genetic damage assessment protocols should be implemented in poultry, with blood samples collected at different stages of embryonic development, not only to coincide with the egg fumigation period, but also to allow monitoring of potential effects over time, minimize the interval between fumigation and sampling, and capture cumulative effects.

From a practical standpoint, FA exhibits antibacterial activity. However, its use is associated with undesirable effects, such as impairments to embryonic development, which can later result in production problems. This indicates that, in production systems, the continuous FA fumigation for sanitizing hatching eggs can increase early chick mortality, compromise flock performance, and result in significant economic losses, in addition to posing occupational risks to workers who are continuously exposed to the product. This necessitates a reconsideration of the use of FA and the exploration of natural, efficient, and safe alternatives to address these issues.

## 4. Conclusions

Common recommendations for the fumigation of hatching eggs in both commercial and non-commercial settings aim primarily to minimize microbial contamination, especially bacterial contamination, and to ensure poultry productivity through high HI rates. At best, the protocols used in this study demonstrated that FA reduced the bacterial load on the eggshell surface without having any antibacterial effect on the embryos. Severe shell damage and decreased HI rates were observed with increasing concentrations of FA. Tracheal lesions in embryos were identified regardless of the concentration fumigated, further highlighting the respiratory risks associated with FA and confirming its potential toxicity. These findings suggest that poultry deaths after hatching may be related to respiratory system damage caused during the embryonic phase by FA fumigation during the pre-incubation period. Finally, the fumigation at 10 g/m^3^ of FA is clearly inadvisable under the evaluated conditions. Even at lower levels (2.5 g/m^3^), caution is recommended. In-depth investigations on the fumigation of hatching eggs with FA are needed, encompassing potential genotoxic effects, comprehensive post-hatching analyses, and further research aimed at safe alternatives.

## Figures and Tables

**Table 1:** Management of formaldehyde (FA) fumigation in hatching eggs.

Treatment	Concentration	Fumigation Time	Fumigation Temperature	Fumigation Humidity	Number of Eggs
Control	.	.	24–26 °C	73–79%	350
FA-I	2.5 g/m^3^	15 min	24–26 °C	73–79%	350
FA-II	5 g/m^3^	15 min	24–26 °C	73–79%	350
FA-III	10 g/m^3^	15 min	24–26 °C	73–79%	350

FA fumigation was performed at 2.5 g/m^3^ (FA-I), 5 g/m^3^ (FA-II), and 10 g/m^3^ (FA-III).

**Table 2:** Bacterial counts on eggshell surfaces and yolk sacs of eggs subjected to different levels of formaldehyde (FA) fumigation.

Treatment	Eggshells	
	TAMB (log_10_ CFU/mL)	ENT
Control	1.57 ± 0.49 ^a^	<10 CFU/mL
FA-I	<10 CFU/mL ^b^	<10 CFU/mL
FA-II	<10 CFU/mL ^b^	<10 CFU/mL
FA-III	<10 CFU/mL ^b^	<10 CFU/mL
*p* value	<0.0009	
**Treatment**	**Yolk sacs**	
	**TAMB (log_10_ CFU/mL)**	**ENT**
Control	2.40 ± 0.22 ^a^	<10 CFU/mL
FA-I	1.85 ± 0.33 ^a^	<10 CFU/mL
FA-II	1.78 ± 0.16 ^a^	<10 CFU/mL
FA-III	1.77 ± 0.43 ^a^	<10 CFU/mL
*p* value	0.0964	

^a,b^ Different letters in the same column mean significant differences using the Tukey test (*p* < 0.05). FA fumigation at 2.5 g/m^3^ (FA-I), 5 g/m^3^ (FA-II), and 10 g/m^3^ (FA-III); TAMB, total aerobic mesophilic bacteria; ENT, Enterobacteriaceae.

**Table 3:** Correlation between the analyzed variables.

	YTAMB	HI	EED	IED	LED	CE
ETAMB	0.52 ns	0.10 ns	0.25 ns	−0.16 ns	0.08 ns	0.41 ns
YTAMB		0.01 ns	−0.13 ns	−0.17 ns	−0.06 ns	0.67 *
HI			−0.87 **	−0.03 ns	−0.48 ns	−0.54 *
EED				0.21 ns	0.10 ns	0.34 ns
IED					−0.47 ns	−0.24 ns
LED						0.11 ns

ETAMB, total mesophilic aerobic bacteria count on the eggshell; YTAMB, total mesophilic aerobic bacteria count in the yolk sac; HI, hatchability of fertile eggs; EED, early embryonic death; IED, intermediate embryonic death; LED, late embryonic death; CE, number of contaminated eggs; ns, not significant; * *p* < 0.05, ** *p* < 0.0001.

**Table 4:** Incubation yield of eggs subjected to different levels of formaldehyde (FA) fumigation.

Group	EWBS (g)	EWDT (g)	EWL (%)	HI (%)	EED (%)	IED (%)	LED (%)
Control	65.83 ± 0.35 ^a^	57.82 ± 0.29 ^a^	12.17 ± 0.66 ^a^	90.40 ± 3.14 ^ab^	3.27 ± 1.14 ^ab^	0.30 ± 0.60 ^a^	3.57 ± 1.68 ^a^
FA-I	65.57 ± 0.67 ^a^	57.35 ± 1.15 ^a^	12.54 ± 0.93 ^a^	96.05 ± 0.98 ^a^	0.89 ± 1.14 ^b^	0.00 ± 0.00 ^a^	2.68 ± 0.60 ^a^
FA-II	65.32 ± 0.29 ^a^	57.29 ± 0.31 ^a^	12.30 ± 0.45 ^a^	91.74 ± 2.60 ^ab^	4.12 ± 2.15 ^ab^	0.60 ± 1.19 ^a^	2.38 ± 2.57 ^a^
FA-III	64.92 ± 0.51 ^a^	56.53 ± 0.91 ^a^	12.93 ± 0.75 ^a^	88.64 ± 4.11 ^b^	5.95 ± 3.22 ^a^	0.30 ± 0.60 ^a^	3.57 ± 2.38 ^a^
*p* value	0.0980	0.1736	0.4863	0.0223	0.0345	0.7256	0.7628

^a,b^ Different superscript letters within the same column indicate significant differences according to Tukey’s test (*p* < 0.05). ^ab^ Means sharing at least one common letter are not significantly different from each other (*p* > 0.05). FA fumigation at 2.5 g/m^3^ (FA-I), 5 g/m^3^ (FA-II), and 10 g/m^3^ (FA-III); EWBS, egg weight before setting; EWDT, egg weight during transfer; EWL, egg weight loss; HI, hatchability of fertile eggs; EED, early embryonic dead; IED, intermediate embryonic dead; LED, late embryonic dead.

**Table 5:** Analysis of weight and quality of chicks from eggs subjected to different levels of formaldehyde (FA) fumigation.

Treatment	CW (g)	CQ
Control	45.69 ± 0.78 ^a^	9.23 ± 0.95 ^ab^
FA-I	44.63 ± 0.31 ^ab^	9.68 ± 0.62 ^a^
FA-II	44.93 ± 0.56 ^ab^	9.35 ± 0.98 ^ab^
FA-III	43.96 ± 0.81 ^b^	9.10 ± 1.03 ^b^
*p* value	0.0187	0.0339

^a,b^ Different superscript letters within the same column indicate significant differences according to Tukey’s test (*p* < 0.05). ^ab^ Means sharing at least one common letter are not significantly different from each other (*p* > 0.05). FA fumigation at 2.5 g/m^3^ (FA-I), 5 g/m^3^ (FA-II), and 10 g/m^3^ (FA-III); CW, chick weight; CQ, chick quality.

**Table 6:** Analysis of the tracheal tissue of each embryo from eggs subjected to different levels of formaldehyde (FA) fumigation ^1^.

Treatment	SN	ECN	GCH	LI	ECD
Control	1	−	−	−	−
Control	2	−	−	−	−
Control	3	−	−	−	−
Control	4	−	−	−	−
Control	5	−	−	−	−
Control	6	−	−	−	−
FA−I	1	−	+	+	++
FA−I	2	−	+	−	++
FA−I	3	−	+	−	++
FA−I	4	−	+	−	+
FA−I	5	−	+	−	++
FA−I	6	−	+	+	++
FA−II	1	−	+	−	+
FA−II	2	−	+	−	+++
FA−II	3	−	++	−	++
FA−II	4	−	++	−	++
FA−II	5	−	+	+	++
FA−II	6	−	+	+	++
FA−III	1	−	++	−	++
FA−III	2	−	++	+	++
FA−III	3	−	++	+	+++
FA−III	4	−	++	+	+++
FA−III	5	−	++	+	+
FA−III	6	+	+	−	++

^1^ The data are presented in the following intensity categories: absent (−), mild (+), moderate (++), or severe (+++). FA fumigation at 2.5 g/m^3^ (FA-I), 5 g/m^3^ (FA-II), and 10 g/m^3^ (FA-III); SN, sample number; ECN, epithelial cell necrosis; GCH, goblet cell hyperplasia; LI, lymphocytic inflammation; ECD, epithelial cell degeneration.

## Data Availability

The data are contained within the article.
